# Effects of vitamin and omega-3 fatty acid co-supplementation on blood glucose in women with gestational diabetes mellitus: A protocol for systematic review and meta analysis

**DOI:** 10.1097/MD.0000000000026106

**Published:** 2021-05-28

**Authors:** Lei He, Zhiwei Xue, Yijun Liu, Ruixin Chen, Shu Zhou

**Affiliations:** a Department of Gynecology and Obstetrics, West China Second Hospital, Sichuan University, Chengdu; b Key Laboratory of Obstetrics and Gynecologic and Pediatric Diseases and Birth Defects of Ministry of Education, West China Second Hospital, Sichuan University, Chengdu, China.

**Keywords:** gestational diabetes mellitus, meta-analysis, omega-3 fatty acid, protocol, vitamin

## Abstract

**Background:**

There is limited study that has conducted a review investigating the clinical effects of vitamin and omega-3 fatty acid co-supplementation on blood glucose in women with gestational diabetes mellitus (GDM). Therefore, in order to provide new evidence-based medical evidence for clinical treatment, we undertook a systematic review and meta-analysis to assess the effectiveness and safety of vitamin and omega-3 fatty acid co-supplementation on blood glucose in women with GDM.

**Methods:**

This protocol was written following the Preferred Reporting Items for Systematic Reviews and Meta-Analyses Protocols (PRISMA-P) statement guidelines. We will conduct systematic reviews and meta-analyses to identify relevant randomized controlled trials (RCTs) involving vitamin and omega-3 fatty acid co-supplementation on GDM in electronic databases including PubMed, Web of Science, Embase, and the Cochrane Library up to June 2021. Exclusion criteria include observational studies, non-RCTs, review articles, studies with a sample size <50, and studies with insufficient outcome data. The primary outcomes include fasting glucose and insulin. Secondary outcomes are evaluated in a homeostasis model of insulin resistance, total antioxidant capacity, triglycerides, total cholesterol, low-density lipoprotein cholesterol, preterm birth and macrosomia over 4 kg.

**Results:**

The review will add to the existing literature by showing compelling evidence and improved guidance in clinic settings.

**Registration number:**

10.17605/OSF.IO/NSW54.

## 1 Introduction

Gestational diabetes mellitus (GDM) impairs glucose tolerance and insulin resistance at the first onset. It affects about 5 percent of all pregnancies worldwide.^[1]^ GDM is associated with severe maternal and infant complications, including neonatal hypoglycemia, preeclampsia, macrosomia, shoulder dystocia, and maternal and infant complications.^[2,3]^ Furthermore, among women with GDM, the risk of developing type 2 diabetes was reported to increase nearly sevenfold. An increase in inflammatory factors leads to the development of gestation-induced insulin resistance and glucose intolerance. Hyperglycemia in GDM increases oxidative stress through a variety of molecular mechanisms, including activation of protein kinase C and increased production of reactive oxygen species in mitochondria.^[4–6]^

Currently, there is growing interest in using omega-3 fatty acids and vitamin during pregnancy. The basis for this interest is largely the results of epidemiological observations showing a significant inverse relationship between omega-3 fatty acids or alpha-tocopherol levels and pregnancy complications.^[7]^ Supplementation with omega-3 fatty acids is known to improve insulin resistance, impaired glucose homeostasis, and elevated lipid concentrations. In addition, levels of vitamins and omega-3 fatty acids were found to be lower in patients with GDM than in healthy pregnant women. Single vitamin or omega-3 supplementation has shown beneficial effects on biomarkers of inflammation, oxidative stress, and pregnancy outcome.^[8,9]^

To the best of our knowledge, there is limited study that has conducted a review investigating the clinical effects of vitamin and omega-3 fatty acid co-supplementation on blood glucose in women with GDM. Therefore, in order to provide new evidence-based medical evidence for clinical treatment, we undertook a systematic review and meta-analysis to assess the effectiveness and safety of vitamin and omega-3 fatty acid co-supplementation on blood glucose in women with GDM.

## 2 Materials and methods

This protocol was written following the Preferred Reporting Items for Systematic Reviews and Meta-Analyses Protocols (PRISMA-P) statement guidelines.

### 2.1 Searching strategy

This systematic review and meta-analysis has been prospective registered in the Open Science Framework Registry, registration number 10.17605/OSF.IO/NSW54. The PRISMA guidelines, the GRADE system and the Cochrane Handbook will be used to evaluate the quality of published results from all included studies to ensure that the results of our meta-analysis are reliable and veritable. We will conduct systematic reviews and meta-analyses to identify relevant randomized controlled trials (RCTs) involving vitamin and omega-3 fatty acid co-supplementation on GDM in electronic databases including PubMed, Web of Science, Embase, and the Cochrane Library up to June 2021. Ethical approval is not necessary because the present meta-analysis will be performed based on previous published studies.

### 2.2 Eligibility criteria

Included studies are considered eligible if they met the Population, Intervention, Comparator, Outcomes, and Study design criteria as follows:

Population: women with GDM;
Intervention: vitamin and omega-3 fatty acid;
Comparator: without vitamin and omega-3 fatty acid;
Outcomes: The primary outcomes include fasting glucose and insulin. Secondary outcomes are evaluated in a homeostasis model of insulin resistance, total antioxidant capacity, triglycerides, total cholesterol, low-density lipoprotein cholesterol, preterm birth and macrosomia over 4 kg.
Study design: Interventional studies.

Exclusion criteria include observational studies, non-RCTs, review articles, studies with a sample size <50, and studies with insufficient outcome data.

### 2.3 Data extraction

In order to achieve a consistency (at least 80%) of extracted items, the data extractors will extract data from a sample of eligible studies. Results of the pilot extraction will be discussed among review authors and extractors. Two independent reviewers will extract data with a predefined extraction template, which includes the following items: study characteristics such as the first author, publication year, study design, follow-up period; patient demographic details such as patients’ number, average age, and gender ratio. The primary outcomes include fasting glucose and insulin. Secondary outcomes are evaluated in a homeostasis model of insulin resistance, total antioxidant capacity, triglycerides, total cholesterol, low-density lipoprotein cholesterol, preterm birth and macrosomia over 4 kg. The original authors will be contacted to request missing data where necessary. Extracted information will be cross-checked by two independent reviewers. Any disagreements will be discussed and resolved in discussion with a third reviewer.

### 2.4 Statistical analysis and data synthesis

Dichotomous data will be analyzed using risk ratio with 95% confidence intervals, whereas continuous variables will be analyzed using weighted mean differences or standardized mean differences. Pooled analyses will be calculated using fixed-effect models, whereas random-effect models will be applied in case of significant heterogeneity across studies. When no events are observed, 0.5 will be added to both arms of the trial. Statistical heterogeneity will be measured using the I^2^ statistic. Meta-regression analyses will be conducted to estimate the extent to which other covariates may have influenced the treatment effects. Sensitivity analyses will be performed to determine the stability of the overall treatment effects. Additionally, publication bias will be assessed using the Begg adjusted rank correlation test and Egger regression asymmetry test. All *P* values will be 2-tailed, and the statistical significance will be set at .05. Review Manager software (v 5.4; Cochrane Collaboration) was used for the meta-analysis.

### 2.5 Risk of bias and quality assessment

According to the Cochrane Handbook for Systematic Reviews of Interventions, the methodological quality and basis of the included literature will be assessed as follows: randomization, allocation concealment, blind method, selective reporting, group similarity at baseline, incomplete outcome data, compliance, timing of outcome assessments, and intention-to-treat analysis.

### 2.6 Assessment of reporting bias

A funnel plot will be used to assess the existence of reporting bias. We will be evaluated whether asymmetry was due to publication bias or relationship between trial size and effect size.

## 3 Discussion

Women with GDM or pre-diabetes often have reduced glucose tolerance and insulin resistance. To the best of our knowledge, there are limited studies that has conducted a review investigating the clinical effects of vitamin and omega-3 fatty acid co-supplementation on blood glucose in women with GDM. Therefore, in order to provide new evidence-based medical evidence for clinical treatment, we undertook a systematic review and meta-analysis to assess the effectiveness and safety of vitamin and omega-3 fatty acid co-supplementation on blood glucose in women with GDM.

## Author contributions

**Conceptualization:** Yijun Liu.

**Data curation:** Lei He, Zhiwei Xue.

**Formal analysis:** Lei He, Zhiwei Xue.

**Funding acquisition:** Ruixin Chen.

**Investigation:** Lei He, Zhiwei Xue.

**Methodology:** Yijun Liu, Shu Zhou.

**Project administration:** Ruixin Chen, Shu Zhou.

**Resources:** Ruixin Chen, Shu Zhou.

**Software:** Yijun Liu.

**Supervision:** Ruixin Chen, Shu Zhou.

**Validation:** Zhiwei Xue, Yijun Liu.

**Visualization:** Yijun Liu.

**Writing – original draft:** Lei He.

**Writing – review & editing:** Ruixin Chen, Shu Zhou.
